# Review: Central nervous system involvement in mitochondrial disease

**DOI:** 10.1111/nan.12333

**Published:** 2016-07-07

**Authors:** N. Z. Lax, G. S. Gorman, D. M. Turnbull

**Affiliations:** ^1^ The Wellcome Trust Centre for Mitochondrial ResearchInstitute of NeuroscienceNewcastle UniversityNewcastle upon TyneUK

**Keywords:** mitochondrial disease, mitochondrial DNA, neurodegeneration, respiratory chain defects

## Abstract

Mitochondrial respiratory chain defects are an important cause of inherited disorders affecting approximately 1 in 5000 people in the UK population. Collectively these disorders are termed ‘mitochondrial diseases’ and they result from either mitochondrial DNA mutations or defects in nuclear DNA. Although they are frequently multisystem disorders, neurological deficits are particularly common, wide‐ranging and disabling for patients. This review details the manifold neurological impairments associated with mitochondrial disease, and describes the efforts to understand how they arise and progressively worsen in patients with mitochondrial disease. We describe advances in our understanding of disease pathogenesis through detailed neuropathological studies and how this has spurred the development of cellular and animal models of disease. We underscore the importance of continued clinical, molecular genetic, neuropathological and animal model studies to fully characterize mitochondrial diseases and understand mechanisms of neurodegeneration. These studies are instrumental for the next phase of mitochondrial research that has a particular emphasis on finding novel ways to treat mitochondrial disease to improve patient care and quality of life.

## Mitochondria

Mitochondria are the main source of energy, in the form of adenosine triphosphate (ATP), in neurons, but they also play important roles in initiating apoptosis, iron‐sulphur cluster biogenesis and calcium buffering. Mitochondria house the machinery necessary to perform oxidative phosphorylation (OXPHOS) within the inner mitochondrial membrane. This machinery comprises an electron transport chain (ETC) which is made up of four multisubunit complexes (complex I–IV) and two mobile electron carriers (ubiquinone and cytochrome *c*) that are thought to form supercomplexes 1. The ETC allows the transfer of electrons through the complexes and the translocation of protons into the intermembrane space. This proton gradient is harnessed by ATP synthase (complex V) to synthesize ATP.

The OXPHOS system is under dual genetic control of the mitochondrial DNA (mtDNA) and nuclear DNA (nDNA). mtDNA exists as a multiple copy circular double‐stranded 16.6 kb DNA molecule and encodes for 13 polypeptide proteins for the ETC and the necessary RNA machinery [2 ribosomal RNAs (rRNAs) and 22 transfer RNAs (tRNAs)] required for their synthesis within the mitochondria 2. The remaining proteins subunits and assembly factors are encoded by the nDNA and imported into mitochondria. In addition to this, the nDNA encodes for proteins involved in mtDNA maintenance and expression and mitochondrial dynamics, including mitochondrial fission and fusion.

## Mitochondrial genetics and disease

Mitochondrial disorders represent a common group of human genetic diseases that result from a primary defect of mitochondrial OXPHOS. Impaired energy generation due to a failure of OXPHOS can therefore result from a genetic defect arising in either the mtDNA or nDNA. MtDNA mutations may either be in the form of point mutations, such as the most common mtDNA mutation m.3243A>G which affects mt‐tRNA^Leu^, or large‐scale mtDNA rearrangements, including single large‐scale mtDNA deletions associated with Kearns–Sayre syndrome (KSS). MtDNA is strictly inherited through the maternal line 3, and can exist in a state of homoplasmy (comprising solely wild type or mutated mtDNA molecules) or heteroplasmy (mixture of wild type and mutated mtDNA). The heteroplasmy level of mutated mtDNA in cell is important for determining whether there is disruption to OXPHOS that results in a detectable biochemical defect.

The heterogeneous nature of mitochondrial disease means that patients may be affected at any age and with multisystem involvement that does not always correlate with genotype, and so this presents a major diagnostic and treatment challenge for patient management. Certain mtDNA mutations are associated with specific syndromes, however, a single mutation can cause several different phenotypes depending on the segregation of the mutation and heteroplasmy level. Perhaps the best example of this is the m.3243A>G *MT‐TL1* mutation which was first described in relation to the classic mitochondrial encephalopathy with lactic acidosis and stroke‐like episodes (MELAS) syndrome 4. However, the m.3243A>G mutation can also lead to chronic progressive external opthalmoplegia (CPEO) and maternally inherited deafness and diabetes. Current estimates of the prevalence of mtDNA mutations contributing to human disease in the north east of England affect 1 in 5000 individuals 5, 6.

The emergence of next generation sequencing has led to a rapid expansion in the number of nDNA mutations being identified 7. Nuclear encoded DNA defects affecting mtDNA maintenance, OXPHOS assembly and structure and mitochondrial dynamics are predicted to affect approximately 2.9 per 100 000 of the adult general population 5. One of the most common nDNA defects, are mutations in the *POLG* gene that encodes for the sole mtDNA polymerase γ and is responsible for replication of the mitochondrial genome. *POLG* mutations result in a range of different clinical phenotypes either with an early onset, such as in Alpers' syndrome, or late onset as seen in patients with CPEO, myoclonic epilepsy myopathy sensory ataxia (MEMSA) and ataxia neuropathy spectrum (ANS) and these are associated with either accumulation of multiple mtDNA deletions and/or depletion of mtDNA content within individual neurons 8.

## Neurological symptoms and neuropathology

Despite only weighing 2% of total body mass, the brain consumes 20% of oxygen and 50% glucose supplied through delivery from the vasculature and is used to drive aerobic respiration within mitochondria 9. Neurons are heavily dependent on mitochondria for the production of energy for a number of processes underpinning neurotransmission including the regulation of the sodium potassium ATPase pump, regulation of intracellular calcium concentration and exocytosis/recycling of synaptic vesicles 10, 11. Glial cells, such as astrocytes, play important roles in glutamate metabolism, and ion and water homeostasis and are dependent on OXPHOS but also contain stores of glycogen for energy metabolism 12. It is for these reasons, the neuronal cells are especially vulnerable to deficits in energy generation within mitochondria and neurological deficits are the most common symptoms reported in patients with mitochondrial disease and are probably the largest contributor to morbidity and mortality. We discuss the common neurological features and associated neuropathological findings.

### Cerebellar ataxia

Cerebellar ataxia is common in both adult and paediatric patients with mitochondrial disease 13, 14, 15. A recent UK cohort study revealed that out of 345 adult patients with mitochondrial disease, 225 were affected by cerebellar ataxia that is progressive in nature. Ataxia is reported in patients harbouring many different mtDNA mutations, including the common tRNA point mutations *MT‐TL1* and *MT‐TK* and also single large‐scale mtDNA deletions. Cerebellar changes are also reported in patients harbouring nDNA mutations, including *RARS2* 16, *C10orf2* 17 and *POLG* mutations 18. In patients harbouring *POLG* defects, ataxia may be reported as one of the prevailing neurological features and is frequently reported in patients with MEMSA 19, 20 and ANS. The main neuroradiological finding is cerebellar volume loss including progressive cerebellar atrophy and to a lesser extent cerebellar hypoplasia 14, 21.

#### Neuropathology studies

The major neuropathological findings include cerebellar atrophy, microinfarcts, Purkinje cell loss and morphological changes, including axonal torpedoes and abnormal dendritic arborisations, in remaining neurons 13, 22, 23, 24. Patients harbouring *MT‐TL1* show Purkinje cell vulnerability and a high frequency of microinfarcts (or ischaemic‐like lesions) affecting the cerebellar cortex. The aetiology of microinfarcts is not well understood, however, it might be the result of energy deficiency within neurons combined with microvascular abnormalities which lead to localized necrotic cell death 25, 26. The pattern of olivo‐cerebellar pathway vulnerability in patients harbouring *MT‐TK* and *POLG* mutations are very similar. Patients with these genetic defects also demonstrate ischaemic‐like lesions that histologically resemble those seen in *MT‐TL1*. Surviving neurons show a profound loss of complex I subunits indicative of complex I deficiency 13, 17. It is not understood if neurons that harbour such a severe complex I defect are capable of normal neurotransmission and why they do not undergo cell death. While cell loss and synaptic changes are detected in patients harbouring a single large‐scale mtDNA deletion associated with KSS 13, 27, 28, the major neurohistopathological hallmark is that of a myelinopathy in the deep white matter of the cerebellum, which is widespread throughout the CNS, and this is attributed to specific changes to the oligodendrocyte populations 29.

### Extrapyramidal features

Extrapyramidal features, including dystonia, akathisia (motor restlessness), Parkinsonism and tardive dyskinesia, have been described in patients harbouring *POLG* mutations. In 2004, Luoma and colleagues reported the first description of Parkinsonism in a patient with autosomal dominant PEO due to mutated *POLG* 30. As then parkinsonism has been shown to co‐segregate with the *POLG* defect 30, 31, respond to levo‐DOPA treatment 30 and the brains of the patients show reduced dopamine‐uptake in the striatum following DaT scan 32, 33, 34.

Leigh syndrome is a progressive neurodegenerative condition affecting infants owing to mutations affecting mtDNA, including mt‐mRNA and mt‐tRNA mutations, and nDNA defects 35, 36. Clinical symptoms include developmental delay, hypotonia, dystonia and failure to thrive. The clinical course may be varied, however, patients often present with respiratory abnormalities, nystagmus and ataxia. Characteristic neuroradiological features include bilateral symmetric lesions affecting the brain stem and basal ganglia which are likely to contribute to the dystonia in these patients.

#### Neuropathology studies

In idiopathic Parkinson's disease (iPD), complex I deficiency and accumulation of mtDNA deletions have been detected in substantia nigra pars compacta (SNpc) neurons 37, 38. In a recent study, mtDNA deletions were also found in SNpc neurons from patients with *POLG* mutations that were very similar to those seen in iPD 39. Changes in SNpc neurons from patients harbouring *POLG* and *C10orf2* mutations showed neuronal loss without alpha‐synuclein or Lewy bodies, whereas remaining neurons demonstrated a preferential complex I deficiency 40. Another study of patients with *POLG* mutations demonstrated severe nigrostriatal degeneration with decreased mtDNA in remaining neurons 41. In both studies, the degree of nigrostriatal degeneration did not correlate with clinical signs of Parkinsonism.

The neuropathological features of Leigh syndrome include symmetrical vasculo‐necrotic lesions affecting the substania nigra, putamen in the basal ganglia and thalamus or sub‐thalamic nuclei. These lesions are characterized by spongiform changes, and cytotoxic oedema and also increased capillary prominence 42.

### Peripheral neuropathies

Peripheral nerve involvement is frequent in mitochondrial disease, and might define the clinical picture in a number of patients with intergenomic signalling defects causing multiple mtDNA deletions and mtDNA depletion. This includes ANS due to *POLG* mutations where the neuropathy is mainly sensory with a loss of proprioception and vibration sense and touch and pinprick sensory changes with a varying degree of distal muscle weakness. Nerve conduction studies show decreased or absent sensory action potentials and varying motor involvement 43. In almost all patients with mitochondrial neurogastrointestinal encephalopathy, due to mutations in *TYMP*, the neuropathy is predominantly demyelinating with slowed conduction velocities and axonal degeneration and segmental demyelination in biopsied nerves 44, 45. Charcot‐Marie‐Tooth hereditary neuropathy Type 2A (CMT2A) disease is due to mutations in *MFN2*, which encodes a protein involved in mitochondrial dynamics, and this leads to a progressive early onset axonal neuropathy 46. Peripheral neuropathy might also be present in other disorders due to mtDNA mutations, including point mutations m.8344A>G and m.3243A>G, in conjunction with other neurological features 47, 48.

#### Neuropathology studies

Relatively few studies have explored neuropathological features associated with peripheral neuropathy, however, a recent electrophysiological and neuropathological study provided evidence of a sensory neuronopathy in patients with *POLG* mutations, and confirmed a loss of sensory neurons and respiratory chain deficiency in remaining cells in the dorsal root ganglia. In this patient, respiratory chain deficiency was associated with lowered mtDNA content in remaining neurons 43. This may explain the common finding of a loss of fibre tracts from the dorsal column in patients with a peripheral neuropathy 43, 49.

### Epilepsy

Epilepsy may be a presenting feature, such as in myoclonic epilepsy ragged red fibres (MERRF) or MEMSA, or emerge on the background of multiple neurological impairments, such as in MELAS, and seizures may occur in any seismology or classification. A recent study identified that approximately 23.1% of 182 adult patients with genetically‐defined mitochondrial disease in a UK cohort develop epilepsy, and that approximately 34.9% of patients with *MT‐TL1* mutation will develop epilepsy and of these 17.5% also develop stroke‐like episodes. In these patients, epilepsy may be classified as focal motor seizures with or without a loss of consciousness. While in patients harbouring *MT‐TK*, epilepsy affects 92% of patients and is usually progressive myoclonus and generalized tonic clonic seizures 50. Epilepsy occurring in infancy is associated with a particularly poor prognosis with a 50% fatality rate in children 9 months after a diagnosis of epilepsy being made 51. Alpers' syndrome affects neonates and infants who may present with refractory seizures, developmental delay and liver involvement. The epilepsy in these patients are particularly devastating as they may develop status epilepticus, which can be focal or generalized, and from which they may never recover 52. Management of epilepsy in mitochondrial disease is often challenging.

#### Neuropathology

**Figure 1:**
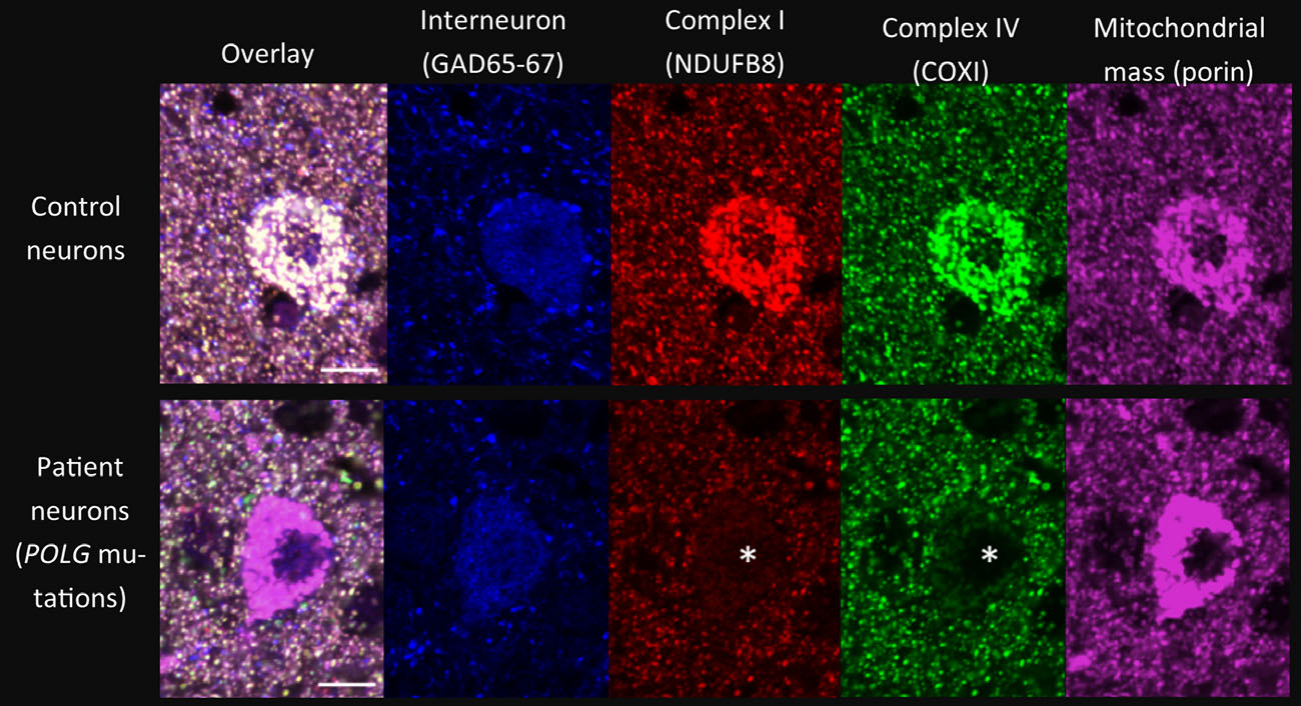
Mitochondrial respiratory chain defects in inhibitory interneurons in a patient with mitochondrial disease. Inhibitory interneurons (GAD65‐67; blue) from a control subject show good co‐localization of a mitochondrial mass marker (porin; magenta) with a subunit of complex I (NDUFB8; red) and complex IV [cytochrome *c* oxidase I (COXI); green]. While inhibitory interneurons from a patient harbouring autosomal recessive mutations in polymerase gamma show decreased expression of both complexes I and IV (marked by white asterix) relative to the mitochondrial mass marker. Scale bar = 10 μm.

It is difficult to pinpoint neuropathological changes specifically associated with seizure activity in patients with mitochondrial disease as there are often multiple neurological impairments coinciding with the epilepsy and it is difficult to dissect primary changes *vs*. secondary changes. Often seizures associated with stroke‐like episodes reveal foci of necrosis affecting the cerebral cortex, and these will be discussed in further detail under ‘seizure‐associated stroke‐like episodes’. The importance of neuronal energy depletion in epilepsy is underscored by the high prevalence of epilepsy in mitochondrial disease. A vulnerability of cortical inhibitory interneurons has recently been shown in post‐mortem tissues from patients with mitochondrial disease with evidence of combined complex I and IV defects and reduced interneuron densities (Figure 1). The identification of combined respiratory chain defects in this study utilizes a novel immunofluorescent assay to interrogate mitochondrial respiratory chain subunit abundance in conjunction with a mitochondrial mass marker within specific neuronal populations, which allows a more precise and accurate method of quantification 53. The combined respiratory chain defect in interneurons is proposed to influence neuronal networks which could contribute to lowered threshold for seizure activity and permit neuronal hyperexcitability but this remains to be substantiated 54. Mitochondrial abnormalities have been detected in both human tissues and animal models of temporal lobe epilepsy with detection of mitochondrial ultrastructural pathology, impairment of complex I in the CA3 region of the hippocampus 55, 56.

### Seizure‐associated stroke‐like episodes

Stroke‐like episodes are often reported in patients with mitochondrial disease and are best defined as an episode of focal cerebral metabolic crisis. Important triggers that have been recognized include febrile illness, headaches, seizures and dehydration. Stroke‐like episodes are one of the main syndromic features of patients with MELAS due to the *MT‐TL1* mutation, and in these patients the standard diagnostic criteria includes clinical features, onset usually before aged 40 years, neuroradiological imaging evidence and seizures 57. However, it not only includes patients with the *MT‐TL1* mutation that are affected by stroke‐like episodes but also includes patients harbouring mutations in *POLG* 58, *FBXL4* 59 and other mtDNA point mutations, including *MT‐TK* mutations 60. In this review, we refer to stroke‐like episodes as seizure‐associated stroke‐like episodes because seizures may signal/be concomitant to the onset of stroke‐like episodes in patients and are often detected on electroencephalogram (EEG) 61.

During the prodromal phase, patients might develop positive visual phenomena including phosphenes and moving objects and often associated with a throbbing headache. As the stroke‐like episodes evolve, patients can develop negative visual phenomena including homonymous hemianopia and cortical blindness; deficits that may become fixed with time. Stroke‐like episodes are detected using magnetic resonance imaging (MRI) as T2‐weighted hyperintensities which are typically in posterior brain regions though not associated with any major vascular territories. Recently there have been a number of radiological studies to determine the mechanisms contributing to the onset of a stroke‐like episode and also to monitor its progression as they can migrate to other regions of the brain. There is evidence that during preclinical or acute onset of a stroke‐like episode, focal hyperperfusion can be detected by either arterial spin labelling or SPECT and can manifest before any structural abnormalities might be seen on MRI 62, 63, 64, 65, 66, 67. In addition to this, there are reports of vasodilation following magnetic resonance angiogram and impaired cerebrovascular reactivity 68, 69. There are conflicting data around apparent diffusion coefficient and whether this is reduced, reflecting cytotoxic oedema, or increased, reflecting vasogenic oedema, in the acute phase of a stroke‐like episode 70, 71, 72, 73, 74. Stroke‐like episodes in mitochondrial disease have a similar manifestation as posterior reversible encephalopathy syndrome (PRES) where headache, encephalopathy, seizures and visual disturbances are common. In PRES, patients typically develop cerebral oedema in the occipital poles, presumed due to cerebral autoregulatory failure 75. Moreover, it has been conjectured that the vulnerability of the posterior circulation to the development of oedema maybe due to the relative reduction in sympathetic innervation of the posterior circulation 75, 76.

The treatment of seizure‐associated stroke‐like episodes involves the urgent control of seizures in the acute phase and then continued anticonvulsants for the prevention of further seizures and development of refractory status epilepticus, a widely recognized complication of mitochondrial disease 77, 78. Additional recent studies advise the implementation of l‐arginine therapy in the acute phase and during the interictal phases of stroke‐like episodes to prevent or decrease the severity of these episodes in patients with MELAS 79. The proposed mechanism of action of l‐arginine is to allow the conversion of l‐arginine into nitric oxide (NO) in the vascular endothelium. NO is a potent mediator of vascular smooth muscle relaxation to improve vasodilation and blood flow in the cerebrovasculature. Recent studies have shown that serum arginine and NO levels are depleted in patients with MELAS and that administration of l‐arginine may be beneficial 80, 81.

There is a clear need for standardized, serial neuroimaging studies of patients with stroke‐like episodes in order to establish specific imaging criteria to accurately detect and monitor stroke‐like episodes, especially when considering therapeutic trials for these patients where accurate and measurable outcomes are crucial for determining drug efficacy. Furthermore, there is an urgent need for effective evidence‐based therapeutic strategies using randomized open‐label, multicentre studies to confirm the efficacy of anticonvulsant drugs and l‐arginine in conjunction with serial neuroimaging to provide insights into the evolution of lesions to the brain and influence the implementation of neuroprotective therapies to prevent the irreversible loss of brain function.

#### Neuropathology

**Figure 2:**
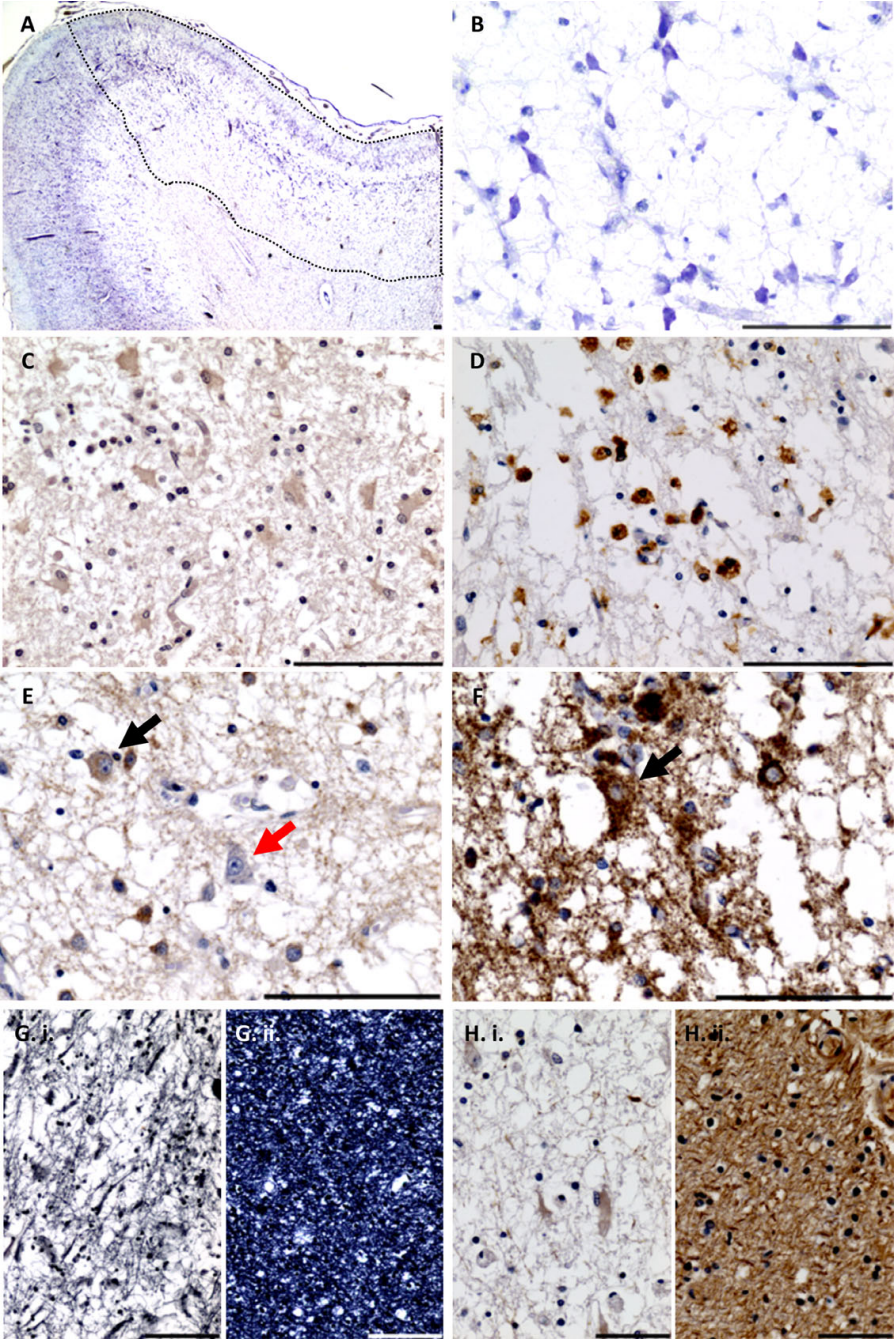
Focal energy‐dependent neuronal necrosis affecting the occipital cortex of a patient harbouring a *MT‐TK* mutation. Focal neuronal necrosis affects the cortical ribbon (**A**; cresyl fast violet) causing neuronal cell loss and spongiform degeneration (**B**; cresyl fast violet) accompanied by astrogliosis (**C**; weak expression of GFAP) and inflammation (**D**; HLA‐DP, DQ, DR). Remaining neurons within the lesion show both complex I expression (**E**; NDUFB8 – black arrow) and complex I deficiency (**E**; NDUFB8 – red arrow), whereas mitochondrial density is maintained in all neurons (**F**; porin – black arrow). Subcortical white matter shows a loss of myelin (**G**.i; Loyez) and axonal loss (**H**.i; phosphorylated neurofilaments), whereas in unaffected cortical regions myelin (**G**.ii; Loyez) and axonal density are maintained (**H**.i; phosphorylated neurofilaments). Scale bar = 100 μm.

The most common neuropathological findings in patients with stroke‐like episodes are the presence of multiple foci of neuronal necrosis (recently termed focal energy‐dependent neuronal necrosis) affecting the posterior cerebral cortex, basal ganglia and cerebellum. These areas of chronic necrosis are ischaemic‐like in appearance; exhibiting neuronal cell loss, destruction of the neuropil and subcortical white matter accompanied by proliferation of astrocytes and inflammatory cells (Figure 2), but they do not conform to major vascular territories. The degree of necrosis varies according to the severity of the stroke‐like episodes, in terms of whether they were recurrent, or timing before death. In some patients, laminar cortical necrosis may be evident affecting cellular layers III and IV–V, whereas in others the entire cortical ribbon may be ablated and might reflect a more chronic process.

**Figure 3:**
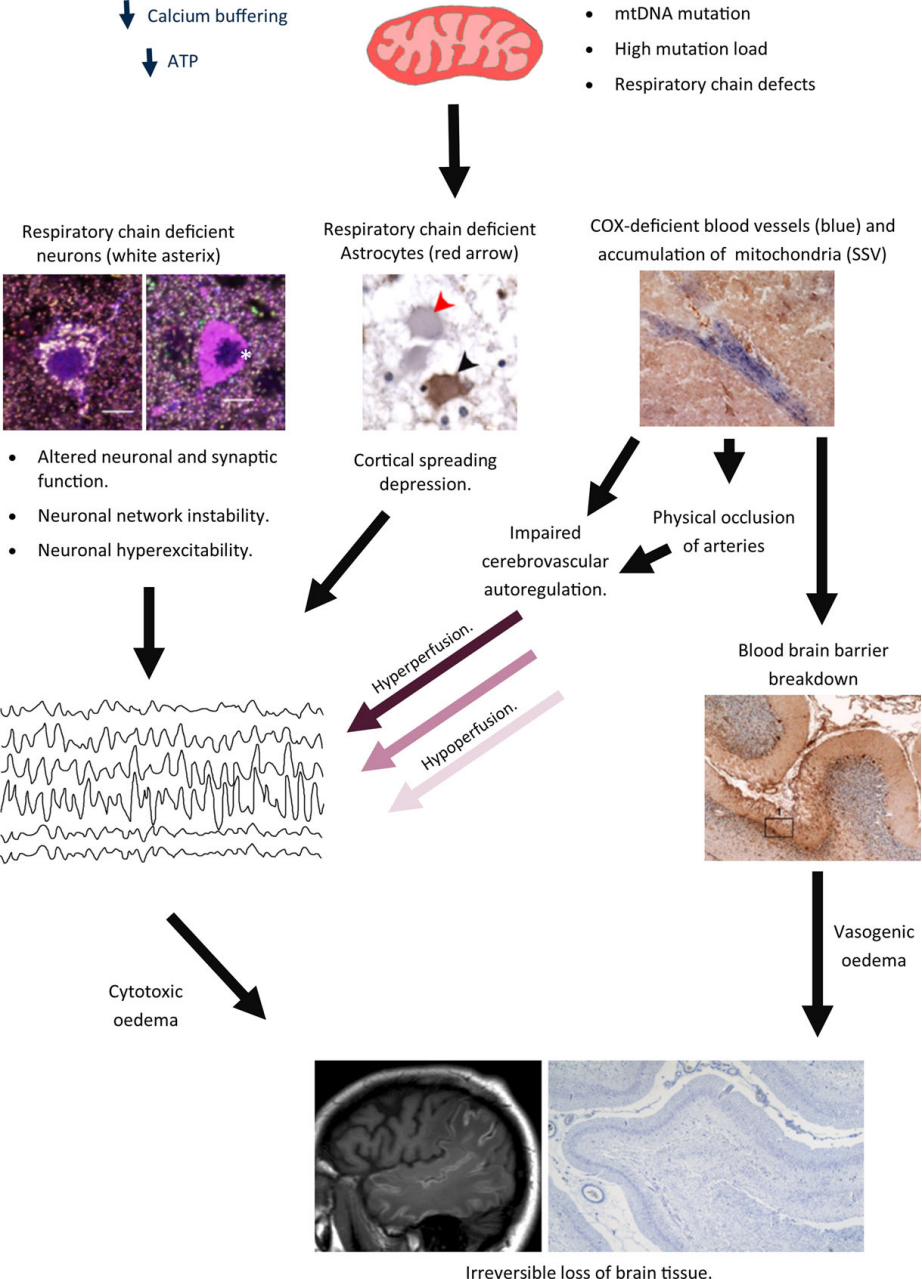
A working hypothesis to dissect out mechanisms underpinning formation of focal neuronal necrotic lesions during stroke‐like episodes. Mitochondrial DNA defects are segregated throughout individual mitochondria and clonally expand to high mutant loads to cause mitochondrial respiratory chain defects defined by a loss of respiratory chain complex activity. Respiratory chain deficiency compromises mitochondrial function leading to reduced adenosine triphosphate (ATP) generation, and impaired calcium buffering. Respiratory chain deficiencies have been documented in multiple cell types within the CNS with neurons, glia and blood vessels (smooth muscle and endothelial cells) and while the impact on cellular function is not yet fully elucidated, the potential mechanisms are described here. Respiratory chain deficient inhibitory interneurons could contribute to modified neuronal and synaptic function leading to neuronal network instability and neuronal hyperexcitability which could in turn lower the threshold for seizure generation. Seizures are commonly reported in the early phase of a stroke‐like episodes and can be detected on electroencephalogram. Respiratory chain deficiencies affecting glial cells, including astrocytes (deficient – red arrow, intact – black arrow), are also observed which could affect synaptic transmission (e.g. glutamate metabolism, ionic homeostasis) and contribute to cortical spreading depression. While respiratory chain deficiencies affecting the CNS vasculature might leading to cerebral auto regulatory failure leading to a mismatch between neuronal activity and appropriate blood flow. This further worsens the ongoing neuronal insult leading to cytotoxic and vascular oedema, hyperintensities on magnetic resonance imaging and irreversible cell loss in necrotic lesions.

While the pathological mechanisms underpinning stroke‐like episodes are not clearly defined, unaffected areas of the brain provide clues with evidence of neuronal respiratory chain defects and angiopathic changes. There are a number of hypotheses to explain mechanisms underpinning neurodegeneration occurring during and after a stroke‐like event, and these include: (i) Mitochondrial cytopathic theory, (ii) Mitochondrial angiopathic theory and finally and (iii) Neuronal hyperexcitability theory. The mitochondrial cytopathic theory suggests that respiratory chain defects and therefore compromised energy production in neurons or glial cells or a combination of both are sufficient to cause focal necrosis 82. The mitochondrial angiopathic theory is supported by evidence of aggregated, enlarged mitochondria within smooth muscle and endothelium in the vasculature, and histochemical data revealing strongly succinate dehydrogenase‐reactive vessels which show an accumulation of mitochondria, similar to ragged red fibres 83, 84. This might represent a morphological abnormality of the microvasculature which leads to physical occlusion. There are contradictory reports which suggest that these abnormal mitochondria still retain cytochrome *c* oxidase (COX) activity, whereas others suggest COX activity is lost and is associated with high mutant load in microvessels 25, 85. In the latter studies, mitochondrial respiratory chain defects affect pial arterioles and smaller penetrating arterioles which might compromise cerebrovascular autoregulation and therefore blood vessel tone. Another proposed mechanism is related to the accumulation of abnormal mitochondria within the endothelium and smooth muscle layers which affects NO‐dependent vasodilatory capacity of the small arteries therefore impeding blood flow. This impaired vasodilation may be corrected through the use of l‐arginine as discussed previously 86. This could lead to areas of the brain with impoverished cerebral blood flow and certain brain areas vulnerable to localized ischaemia. Why this occurs with a posterior predilection is not known, however, the posterior circulation has inadequate sympathetic innervation to mediate autoregulatory control of blood flow 87, this coupled with an energy crisis in arterioles might be a factor in causing focal necrotic lesions. The final theory proposes that neuronal hyperexcitability is responsible for the lesions as seizures are often detected on EEG during a stroke‐like episode, and neuronal hyperexcitability will worsen the energy imbalance between ATP availability and energy requirement, and ultimately lead to neuronal cell death 64, 88. It is likely that the aetiology of the focal necrotic lesions are multifactorial, and probably a combination of all three theories influences the emergence of lesions in the brain (Figure 3).

### Cognitive decline and behavioural disturbance

Cognitive impairment and dementia have been frequently in case reports of patients with mitochondrial disease due to mtDNA mutations, however, there are very few large‐scale longitudinal studies and standardised criteria to define cognitive impairments. A neuropsychological study conducted on 16 adult patients with mitochondrial disease (either due to MT‐TK point mutation, single large‐scale mtDNA deletion or multiple mtDNA deletions) revealed nonverbal cognitive impairments and verbal short‐term memory deficits, associated with working memory, with hypometabolism in the temporal lobes on neuroradiological imaging 89. In a separate study, patients with either a single large‐scale deletion or MT‐TL1 point mutation identified focal deficits in visual construction, attention and abstraction 90. In patients harbouring the MT‐TL1 mutation, neuropsychological testing provided evidence of a global deterioration in cognition affecting executive function, attention, language, memory, visuospatial and motor function 91. Cerebral lactic acidosis in patients harbouring MT‐TL1 mutations correlates with neuropsychological scores in patients with or without stroke‐like episodes implying dysfunction in the frontal domains which is not detected by neuroradiological imaging 92.

#### Neuropathology

There is a paucity of studies correlating neuropathological changes with cognitive impairments in mitochondrial disease. Although it is likely that deterioration in cognition might be attributed to the presence of necrotic foci which impinge on multiple circuits involved in cognitive processing, certainly cognitive decline has been observed in patients without stroke‐like episodes implying that the frontal domains are affected. Neuropathological studies detect changes in neuronal populations in patients affected by either mtDNA or nDNA mutations which could contribute to cognitive dysfunction in mitochondrial disease. A loss of calbindin expression in hippocampal neurons without neuronal loss has been described, and also loss of cortical inhibitory interneurons and respiratory chain deficiencies which could certainly contribute to impaired network activity in the brain 54, 93.

## Modelling the neurology of mitochondrial disease

Clinical, molecular genetic and neuropathological studies have given important insights into the disease pathogenesis. The application of the knowledge gained from these studies is now driving the development of cell and animal model systems to test hypotheses and adopt a functional approach to understanding mechanisms and evaluate the efficacy of treatment.

### Transgenic mouse models

Modelling mtDNA disease has been impeded by the inability to generate stable mtDNA point mutations in mice without undergoing a bottleneck effect and loss of the mutation through successive generations of mice 94. Many of the models generated to date have utilized Cre/Lox technology to selectively knockout (KO) nuclear genes involved in: (i) mtDNA maintenance, replication, transcription, translation (ii) expression and assembly of OXPHOS complexes and (iii) mitochondrial dynamics to evaluate the effect on specific populations of neurons. A comprehensive description of these models is beyond the scope of the current review and we refer readers to a three part review miniseries published last year which discusses each model in detail 95, 96, 97.

Here, we provide a brief overview and examples of models associated with defects in; (i) mtDNA maintenance and (ii) OXPHOS in certain neuronal populations. A number of mouse models have been generated by targeting *TFAM* which is a nuclear encoded transcriptional activator which has a number of roles within the mitochondrion including binding to the mtDNA promotor to activate mitochondrial transcription, provides RNA primers to facilitate mtDNA replication and also plays a histone‐like role by coating the mtDNA 98. There are two models which have used neuronal‐specific KO of *TFAM*, including forebrain neurons using a CaMKIIα‐Cre [Mitochondrial Late Onset Neurodegeneration (MILON) mice] and dopaminergic neurons using a DAT‐Cre (MitoPark mice) which lead mtDNA depletion in the targeted neurons. The MILON mice show a reduction in mtDNA copy number in neocortex at 2 months of age and develop a phenotype at age 5–6 months whereby they severely deteriorate and die within 2–3 weeks after onset 99. Neuropathologically the neocortex undergoes severe degeneration and loss of organization, whereas in the hippocampus, CA1 is absent with a profound loss of pyramidal cells, CA2 is intact and CA3 is slightly affected. Chimeric MILON mice were generated to study the effects of mosaic pattern of wild type and mtDNA depletion in forebrain neurons. They revealed that low levels (20%) of respiratory chain deficiency were sufficient to produce behavioural abnormalities, whereas higher levels (80%) of respiratory chain deficiency were sufficient to induce trans‐neuronal degeneration events 100.

The MitoPark mice harbour dopaminergic neuron‐specific KO of *TFAM* and develop a progressive Parkinson‐like phenotype at 12 weeks of age with motor deficits, dopamine depletion and SNpc degeneration 101. In asymptomatic mice aged 6–8 weeks, electrophysiological experiments reveal subtle changes in nigrostriatal function with an absence of pacemaker activity in SNpc neurons and impaired dopamine release from their axonal terminals 102.

The second group of transgenic mouse models we discuss are those associated with defects in OXPHOS either due to abolished complex subunit or assembly unit expression. There are a number of model which target subunits associated with complex I as complex I deficiencies are frequently detected in mitochondrial disease. A neuronal and glial‐specific KO (using a nestin‐Cre) of *NDUFS4* (*NADH:ubiquinone oxidoreductase subunit S4*), which encodes for a nuclear‐encoded accessory subunit of complex I, has been generated. This mouse shows rapidly progressive gait impairments, breathing difficulties and death by 7 weeks of age and therefore resembles Leigh syndrome. Neuropathological features include complex I deficiency and progressive degeneration in the form of spongiosis and lesions within the olfactory bulb, cerebellum and vestibular nuclei accompanied by progressive glial activation and inflammation 103, 104. A recent study has investigated hypoxia as a possible treatment strategy for ameliorating disease progression; *NDUFS4* KO mice were exposed to chronic hypoxia (11% oxygen) environment showed extended lifespan with improved motor coordination and reduced neuroinflammation upon neurohistopathological assessment 105.

Mice harbouring *NDUFA5* (*NADH:ubiquinone oxidoreductase subunit A5*) KO, a nuclear‐encoded structural subunit of complex I, in forebrain neurons (using CaMKIIα‐Cre) were healthy until 10–11 months of age when they became lethargic, demonstrated a loss of motor coordination and hindlimb clasping. These mice showed only a mild encephalopathy with evidence of complex I deficient neurons without evidence of neurodegeneration which the authors suggest might be related to compensatory processes 106.

Recently, forebrain neuron‐specific (using CaMKIIα‐Cre) KOs for *RISP* (encoding for a catalytic subunit of complex III; *Reiske iron‐sulphur cluster protein*) and *COX10* (encoding an accessory protein involved in assembly of COX) have been created to understand more about the selective brain involvement in mitochondrial disease. These studies revealed that the *RISP*‐KO mice developed a rapid phenotype aged 2 months with poor rotarod performance with a rapid disease progression and died aged 3–3.5 months. Evaluation of CNS tissues confirmed complex III deficiency and high levels of oxidative stress in remaining neurons, particularly affecting the piriform cortex. While in the *COX10*‐KO mice, rotarod performance was poor aged 3 months but they survived until aged 8–12 months with a vulnerability of the cingulate cortex and oxidative stress in remaining neurons 107.

### Induced pluripotent stem cells

Induced pluripotent stem cells (iPSC) hold the potential for *in vitro* disease modelling, drug screening and cell‐replacement therapies. iPSCs constitute cells which are capable of self‐renewal and can differentiate into any cell type and are generated by reprogramming patient fibroblasts with pluriopotency associated transcription factors. In recent years, a number of studies have utilized patient‐derived iPSC to understand mechanisms of mitochondrial disease exploiting the main advantages of (i) maintaining the nuclear genetic background of the patient, and (ii) differentiation into disease‐specific tissue‐ or cell‐type, e.g. postmitotic neurons. There are some caveats associated with the study of iPSCs including the acquisition of genome instability during culture 108, the alteration of mitochondrial morphology with rounded appearance and poor cristae structure, low mtDNA copy number and respiratory capacity to facilitate the metabolic switch to glycolysis. In addition, impaired mtDNA integrity may be a by‐product of cellular reprogramming 109. There have been a number of studies utilizing iPSCs to understand pathogenetic mechanisms in mitochondrial disease, and these are discussed in more detail in a recent review by Hatakeyama and colleagues 110.

Induced pluripotent stem cells have been derived from fibroblasts from patients with MELAS due to the m.3243A>G mutation and two cell lines were generated; one harbouring high heteroplasmy levels (>80%) and the other which was low heteroplasmy levels (undetectable). Following differentiation into neurons, the high heteroplasmy line showed downregulation of complex I and an increase in mitophagy 111. Another study that investigated fibroblasts from a patient with MELAS created iPSC harbouring the m.13513G>A mutation at heteroplasmic levels (50–60%) and a mutation‐free cell line. Heteroplasmic cell lines showed a decline in heteroplasmy levels with long‐term maintenance in culture 112. In a recent study, cellular homoplasmy for the m.8993T>A mutation associated with Leigh syndrome was genetically corrected using somatic cell nuclear transfer 113.

## Future directions/studies

Unlike other neurodegenerative diseases, brains from patients with mitochondrial disease show atrophy and severe neuronal cell loss that this is not associated with any extra‐ or intracellular accumulation of misfolded proteins. The salient neuropathological features are the decrease or absence of mitochondrial respiratory chain subunits, particularly involving subunits comprising complex I, and high ratio mutated mtDNA to wild type mtDNA in surviving neurons. The future of mitochondrial research should aim to prevent the irreversible brain loss and neurological disability in these patients. Post‐mortem human brain studies are invaluable for characterizing the chronic neurodegenerative changes, and new techniques, such as CLARITY 114, 115, will dramatically improve our understanding of disease pathogenesis. However, our neuropathological studies can be strengthened by linking to patient iPSCs and transgenic mouse models to interrogate functional systems and enhance our understanding of progressive mechanisms of degeneration and facilitate the development of better‐directed therapeutic intervention.

## References

[1] Lapuente‐Brun E , Moreno‐Loshuertos R , Acin‐Perez R , Latorre‐Pellicer A , Colas C , Balsa E , Perales‐Clemente E , Quirós PM , Calvo E , Rodríguez‐Hernández MA , Navas P , Cruz R , Carracedo Á , López‐Otín C , Pérez‐Martos A , Fernández‐Silva P , Fernández‐Vizarra E , Enríquez JA . Supercomplex assembly determines electron flux in the mitochondrial electron transport chain. Science 2013; 340: 1567–70 23812712 10.1126/science.1230381

[2] Anderson S , Bankier AT , Barrell BG , de Bruijn MH , Coulson AR , Drouin J , Eperon IC , Nierlich DP , Roe BA , Sanger F , Schreier PH , Smith AJ , Staden R , Young IG . Sequence and organization of the human mitochondrial genome. Nature 1981; 290: 457–65 7219534 10.1038/290457a0

[3] Sutovsky P , Moreno RD , Ramalho‐Santos J , Dominko T , Simerly C , Schatten G . Ubiquitin tag for sperm mitochondria. Nature 1999; 402: 371–2 10586873 10.1038/46466

[4] Pavlakis SG , Phillips PC , DiMauro S , De Vivo DC , Rowland LP . Mitochondrial myopathy, encephalopathy, lactic acidosis, and strokelike episodes: a distinctive clinical syndrome. Ann Neurol 1984; 16: 481–8 6093682 10.1002/ana.410160409

[5] Gorman GS , Schaefer AM , Ng Y , Gomez N , Blakely EL , Alston CL , Feeney C , Horvath R , Yu‐Wai‐Man P , Chinnery PF , Taylor RW , Turnbull DM , McFarland R . Prevalence of nuclear and mitochondrial DNA mutations related to adult mitochondrial disease. Ann Neurol 2015; 77: 753–9 25652200 10.1002/ana.24362 PMC4737121

[6] Schaefer AM , McFarland R , Blakely EL , He L , Whittaker RG , Taylor RW , Chinnery PF , Turnbull DM .Prevalence of mitochondrial DNA disease in adults. Ann Neurol 2008; 63: 35–9 17886296 10.1002/ana.21217

[7] Lightowlers RN , Taylor RW , Turnbull DM . Mutations causing mitochondrial disease: what is new and what challenges remain? Science 2015; 349: 1494–9 26404827 10.1126/science.aac7516

[8] Horvath R , Hudson G , Ferrari G , Futterer N , Ahola S , Lamantea E , Prokisch H , Lochmüller H , McFarland R , Ramesh V , Klopstock T , Freisinger P , Salvi F , Mayr JA , Santer R , Tesarova M , Zeman J , Udd B , Taylor RW , Turnbull D , Hanna M , Fialho D , Suomalainen A , Zeviani M , Chinnery PF . Phenotypic spectrum associated with mutations of the mitochondrial polymerase gamma gene. Brain 2006; 129: 1674–84 16621917 10.1093/brain/awl088

[9] Attwell D , Laughlin SB . An energy budget for signaling in the grey matter of the brain. J Cereb Blood Flow Metab 2001; 21: 1133–45 11598490 10.1097/00004647-200110000-00001

[10] Rangaraju V , Calloway N , Ryan TA . Activity‐driven local ATP synthesis is required for synaptic function. Cell 2014; 156: 825–35 24529383 10.1016/j.cell.2013.12.042 PMC3955179

[11] Vos M , Lauwers E , Verstreken P . Synaptic mitochondria in synaptic transmission and organization of vesicle pools in health and disease. Front Synaptic Neurosci 2010; 2: 139 21423525 10.3389/fnsyn.2010.00139 PMC3059669

[12] Hertz L , Peng L , Dienel GA . Energy metabolism in astrocytes: high rate of oxidative metabolism and spatiotemporal dependence on glycolysis/glycogenolysis. J Cereb Blood Flow Metab 2007; 27: 219–49 16835632 10.1038/sj.jcbfm.9600343

[13] Lax NZ , Hepplewhite PD , Reeve AK , Nesbitt V , McFarland R , Jaros E , Taylor RW , Turnbull DM . Cerebellar ataxia in patients with mitochondrial DNA disease: a molecular clinicopathological study. J Neuropathol Exp Neurol 2012; 71: 148–61 22249460 10.1097/NEN.0b013e318244477d PMC3272439

[14] Scaglia F , Wong LJ , Vladutiu GD , Hunter JV . Predominant cerebellar volume loss as a neuroradiologic feature of pediatric respiratory chain defects. AJNR Am J Neuroradiol 2005; 26: 1675–80 16091512 PMC7975170

[15] Bargiela D , Shanmugarajah P , Lo C , Blakely EL , Taylor RW , Horvath R , Wharton S , Chinnery PF , Hadjivassiliou M . Mitochondrial pathology in progressive cerebellar ataxia. Cerebellum Ataxias 2015; 2: 16 26640698 10.1186/s40673-015-0035-x PMC4670505

[16] Joseph JT , Innes AM , Smith AC , Vanstone MR , Schwartzentruber JA , Bulman DE , Majewski J , Daza RA , Hevner RF , Michaud J , Boycott KM ; FORGE Canada Consortium . Neuropathologic features of pontocerebellar hypoplasia type 6. J Neuropathol Exp Neurol 2014; 73: 1009–25 25289895 10.1097/NEN.0000000000000123

[17] Hakonen AH , Goffart S , Marjavaara S , Paetau A , Cooper H , Mattila K , Lampinen M , Sajantila A , Lönnqvist T , Spelbrink JN , Suomalainen A . Infantile‐onset spinocerebellar ataxia and mitochondrial recessive ataxia syndrome are associated with neuronal complex I defect and mtDNA depletion. Hum Mol Genet 2008; 17: 3822–35 18775955 10.1093/hmg/ddn280

[18] Synofzik M , Srulijes K , Godau J , Berg D , Schols L . Characterizing POLG ataxia: clinics, electrophysiology and imaging. Cerebellum 2012; 11: 1002–11 22528963 10.1007/s12311-012-0378-2

[19] Hakonen AH , Heiskanen S , Juvonen V , Lappalainen I , Luoma PT , Rantamaki M , Goethem GV , Lofgren A , Hackman P , Paetau A , Kaakkola S , Majamaa K , Varilo T , Udd B , Kaariainen H , Bindoff LA , Suomalainen A . Mitochondrial DNA polymerase W748S mutation: a common cause of autosomal recessive ataxia with ancient European origin. Am J Hum Genet 2005; 77: 430–41 16080118 10.1086/444548 PMC1226208

[20] Mignarri A , Cenciarelli S , Da Pozzo P , Cardaioli E , Malandrini A , Federico A , Dotti MT . Mitochondrial recessive ataxia syndrome: a neurological rarity not to be missed. J Neurol Sci 2015; 349: 254–5 25586537 10.1016/j.jns.2014.12.040

[21] Tschampa HJ , Urbach H , Greschus S , Kunz WS , Kornblum C . Neuroimaging characteristics in mitochondrial encephalopathies associated with the m.3243A>G MTTL1 mutation. J Neurol 2013; 260: 1071–80 23196335 10.1007/s00415-012-6763-4

[22] Mori O , Yamazaki M , Ohaki Y , Arai Y , Oguro T , Shimizu H , Asano G . Mitochondrial encephalomyopathy with lactic acidosis and stroke like episodes (MELAS) with prominent degeneration of the intestinal wall and cactus‐like cerebellar pathology. Acta Neuropathol (Berl) 2000; 100: 712–17 11078225 10.1007/s004010000209

[23] Sparaco M , Simonati A , Cavallaro T , Bartolomei L , Grauso M , Piscioli F , Morelli L , Rizzuto N . MELAS: clinical phenotype and morphological brain abnormalities. Acta Neuropathol (Berl) 2003; 106: 202–12 12910360 10.1007/s00401-003-0716-z

[24] Tanahashi C , Nakayama A , Yoshida M , Ito M , Mori N , Hashizume Y . MELAS with the mitochondrial DNA 3243 point mutation: a neuropathological study. Acta Neuropathol (Berl) 2000; 99: 31–8 10651025 10.1007/pl00007403

[25] Lax NZ , Pienaar IS , Reeve AK , Hepplewhite PD , Jaros E , Taylor RW , Kalaria RN , Turnbull DM . Microangiopathy in the cerebellum of patients with mitochondrial DNA disease. Brain 2012; 135: 1736–50 22577219 10.1093/brain/aws110 PMC3359757

[26] Tzoulis C , Neckelmann G , Mork SJ , Engelsen BE , Viscomi C , Moen G , Ersland L , Zeviani M , Bindoff LA . Localized cerebral energy failure in DNA polymerase gamma‐associated encephalopathy syndromes. Brain 2010; 133: 1428–37 20400524 10.1093/brain/awq067

[27] Tanji K , DiMauro S , Bonilla E . Disconnection of cerebellar Purkinje cells in Kearns‐Sayre syndrome. J Neurol Sci 1999; 166: 64–70 10465502 10.1016/s0022-510x(99)00114-8

[28] Chrysostomou A , Grady JP , Laude A , Taylor RW , Turnbull DM , Lax NZ . Investigating complex I deficiency in Purkinje cells and synapses in patients with mitochondrial disease. Neuropathol Appl Neurobiol 2015; doi:10.1111/nan.12282 10.1111/nan.12282 PMC4973693 26337858

[29] Lax NZ , Campbell GR , Reeve AK , Ohno N , Zambonin J , Blakely EL , Taylor RW , Bonilla E , Tanji K , DiMauro S , Jaros E , Lassmann H , Turnbull DM , Mahad DJ . Loss of myelin‐associated glycoprotein in kearns‐sayre syndrome. Arch Neurol 2012; 69: 490–9 22491194 10.1001/archneurol.2011.2167 PMC3672633

[30] Luoma P , Melberg A , Rinne JO , Kaukonen JA , Nupponen NN , Chalmers RM , Oldfors A , Rautakorpi I , Peltonen L , Majamaa K , Somer H , Suomalainen A . Parkinsonism, premature menopause, and mitochondrial DNA polymerase gamma mutations: clinical and molecular genetic study. Lancet 2004; 364: 875–82 15351195 10.1016/S0140-6736(04)16983-3

[31] Hudson G , Schaefer AM , Taylor RW , Tiangyou W , Gibson A , Venables G , Griffiths P , Burn DJ , Turnbull DM , Chinnery PF . Mutation of the linker region of the polymerase gamma‐1 (POLG1) gene associated with progressive external ophthalmoplegia and Parkinsonism. Arch Neurol 2007; 64: 553–7 17420318 10.1001/archneur.64.4.553

[32] Bandettini di Poggio M , Nesti C , Bruno C , Meschini MC , Schenone A , Santorelli FM . Dopamine‐agonist responsive Parkinsonism in a patient with the SANDO syndrome caused by POLG mutation. BMC Med Genet 2013; 14: 105 24099403 10.1186/1471-2350-14-105 PMC3851930

[33] Miguel R , Gago MF , Martins J , Barros P , Vale J , Rosas MJ . POLG1‐related levodopa‐responsive parkinsonism. Clin Neurol Neurosurg 2014; 126: 47–54 25203713 10.1016/j.clineuro.2014.08.020

[34] Tzoulis C , Schwarzlmuller T , Biermann M , Haugarvoll K , Bindoff LA . Mitochondrial DNA homeostasis is essential for nigrostriatal integrity. Mitochondrion 2016; 28: 33–7 26979109 10.1016/j.mito.2016.03.003

[35] Rahman S , Blok RB , Dahl HH , Danks DM , Kirby DM , Chow CW , Christodoulou J , Thorburn DR . Leigh syndrome: clinical features and biochemical and DNA abnormalities. Ann Neurol 1996; 39: 343–51 8602753 10.1002/ana.410390311

[36] Zhu Z , Yao J , Johns T , Fu K , De Bie I , Macmillan C , Cuthbert AP , Newbold RF , Wang J , Chevrette M , Brown GK , Brown RM , Shoubridge EA . SURF1, encoding a factor involved in the biogenesis of cytochrome *c* oxidase, is mutated in Leigh syndrome. Nat Genet 1998; 20: 337–43 9843204 10.1038/3804

[37] Bender A , Krishnan KJ , Morris CM , Taylor GA , Reeve AK , Perry RH , Jaros E , Hersheson JS , Betts J , Klopstock T , Taylor RW , Turnbull DM . High levels of mitochondrial DNA deletions in substantia nigra neurons in aging and Parkinson disease. Nat Genet 2006; 38: 515–17 16604074 10.1038/ng1769

[38] Kraytsberg Y , Kudryavtseva E , McKee AC , Geula C , Kowall NW , Khrapko K . Mitochondrial DNA deletions are abundant and cause functional impairment in aged human substantia nigra neurons. Nat Genet 2006; 38: 518–20 16604072 10.1038/ng1778

[39] Reeve AK , Park TK , Jaros E , Campbell GR , Lax NZ , Hepplewhite PD , Krishnan KJ , Elson JL , Morris CM , McKeith IG , Turnbull DM . Relationship between mitochondria and alpha‐synuclein: a study of single substantia nigra neurons. Arch Neurol 2012; 69: 385–93 22410447 10.1001/archneurol.2011.2675

[40] Palin EJ , Paetau A , Suomalainen A . Mesencephalic complex I deficiency does not correlate with parkinsonism in mitochondrial DNA maintenance disorders. Brain 2013; 136: 2379–92 23811324 10.1093/brain/awt160

[41] Tzoulis C , Tran GT , Schwarzlmuller T , Specht K , Haugarvoll K , Balafkan N , Lilleng PK , Miletic H , Biermann M , Bindoff LA . Severe nigrostriatal degeneration without clinical parkinsonism in patients with polymerase gamma mutations. Brain 2013; 136: 2393–404 23625061 10.1093/brain/awt103

[42] Leigh D . Subacute necrotizing encephalomyelopathy in an infant. J Neurol Neurosurg Psychiatry 1951; 14: 216–21 14874135 10.1136/jnnp.14.3.216 PMC499520

[43] Lax NZ , Whittaker RG , Hepplewhite PD , Reeve AK , Blakely EL , Jaros E , Ince PG , Taylor RW , Fawcett PR , Turnbull DM . Sensory neuronopathy in patients harbouring recessive polymerase gamma mutations. Brain 2012; 135: 62–71 22189570 10.1093/brain/awr326 PMC3267986

[44] Bedlack RS , Vu T , Hammans S , Sparr SA , Myers B , Morgenlander J , Hirano M . MNGIE neuropathy: five cases mimicking chronic inflammatory demyelinating polyneuropathy. Muscle Nerve 2004; 29: 364–8 14981734 10.1002/mus.10546

[45] Said G , Lacroix C , Plante‐Bordeneuve V , Messing B , Slama A , Crenn P , Nivelon‐Chevallier A , Bedenne L , Soichot P , Manceau E , Rigaud D , Guiochon‐Mantel A , Matuchansky C . Clinicopathological aspects of the neuropathy of neurogastrointestinal encephalomyopathy (MNGIE) in four patients including two with a Charcot‐Marie‐Tooth presentation. J Neurol 2005; 252: 655–62 15742109 10.1007/s00415-005-0712-4

[46] Feely SM , Laura M , Siskind CE , Sottile S , Davis M , Gibbons VS , Reilly MM , Shy ME . MFN2 mutations cause severe phenotypes in most patients with CMT2A. Neurology 2011; 76: 1690–6 21508331 10.1212/WNL.0b013e31821a441e PMC3100135

[47] Karppa M , Syrjala P , Tolonen U , Majamaa K . Peripheral neuropathy in patients with the 3243A>G mutation in mitochondrial DNA. J Neurol 2003; 250: 216–21 12574954 10.1007/s00415-003-0981-8

[48] Kaufmann P , Pascual JM , Anziska Y , Gooch CL , Engelstad K , Jhung S , DiMauro S , De Vivo DC . Nerve conduction abnormalities in patients with MELAS and the A3243G mutation. Arch Neurol 2006; 63: 746–8 16682545 10.1001/archneur.63.5.746

[49] Hopkins SE , Somoza A , Gilbert DL . Rare autosomal dominant POLG1 mutation in a family with metabolic strokes, posterior column spinal degeneration, and multi‐endocrine disease. J Child Neurol 2010; 25: 752–6 19815814 10.1177/0883073809343313

[50] Whittaker RG , Devine HE , Gorman GS , Schaefer AM , Horvath R , Ng Y , Nesbitt V , Lax NZ , McFarland R , Cunningham MO , Taylor RW , Turnbull DM . Epilepsy in adults with mitochondrial disease: a cohort study. Ann Neurol 2015; 78: 949–57 26381753 10.1002/ana.24525 PMC4737309

[51] El Sabbagh S , Lebre AS , Bahi‐Buisson N , Delonlay P , Soufflet C , Boddaert N , Rio M , Rötig A , Dulac O , Munnich A , Desguerre I . Epileptic phenotypes in children with respiratory chain disorders. Epilepsia 2010; 51: 1225–35 20196775 10.1111/j.1528-1167.2009.02504.x

[52] Nguyen KV , Ostergaard E , Ravn SH , Balslev T , Danielsen ER , Vardag A , McKiernan PJ , Gray G , Naviaux RK . POLG mutations in Alpers syndrome. Neurology 2005; 65: 1493–5 16177225 10.1212/01.wnl.0000182814.55361.70

[53] Grunewald A , Lax NZ , Rocha MC , Reeve AK , Hepplewhite PD , Rygiel KA , Taylor RW , Turnbull DM . Quantitative quadruple‐label immunofluorescence of mitochondrial and cytoplasmic proteins in single neurons from human midbrain tissue. J Neurosci Methods 2014; 232: 143–9 24880043 10.1016/j.jneumeth.2014.05.026 PMC4076514

[54] Lax NZ , Grady J , Laude A , Chan F , Hepplewhite PD , Gorman G , Whittaker RG , Ng Y , Cunningham MO , Turnbull DM . Extensive respiratory chain defects in inhibitory interneurones in patients with mitochondrial disease. Neuropathol Appl Neurobiol 2015; 42: 180–93 25786813 10.1111/nan.12238 PMC4772453

[55] Kunz WS , Kudin AP , Vielhaber S , Blumcke I , Zuschratter W , Schramm J , Beck H , Elger CE . Mitochondrial complex I deficiency in the epileptic focus of patients with temporal lobe epilepsy. Ann Neurol 2000; 48: 766–73 11079540

[56] Simeone KA , Matthews SA , Samson KK , Simeone TA . Targeting deficiencies in mitochondrial respiratory complex I and functional uncoupling exerts anti‐seizure effects in a genetic model of temporal lobe epilepsy and in a model of acute temporal lobe seizures. Exp Neurol 2014; 251: 84–90 24270080 10.1016/j.expneurol.2013.11.005 PMC3990438

[57] Hirano M , Ricci E , Koenigsberger MR , Defendini R , Pavlakis SG , DeVivo DC , DiMauro S , Rowland LP . Melas: an original case and clinical criteria for diagnosis. Neuromuscul Disord 1992; 2: 125–35 1422200 10.1016/0960-8966(92)90045-8

[58] Deschauer M , Tennant S , Rokicka A , He L , Kraya T , Turnbull DM , Zierz S , Taylor RW . MELAS associated with mutations in the POLG1 gene. Neurology 2007; 68: 1741–2 17502560 10.1212/01.wnl.0000261929.92478.3e

[59] Ebrahimi‐Fakhari D , Seitz A , Kolker S , Hoffmann GF . Recurrent stroke‐like episodes in FBXL4‐associated early‐onset mitochondrial encephalomyopathy. Pediatr Neurol 2015; 53: 549–50 26421988 10.1016/j.pediatrneurol.2015.08.018

[60] Tanji K , Gamez J , Cervera C , Mearin F , Ortega A , de la Torre J , Montoya J , Andreu AL , DiMauro S , Bonilla E . The A8344G mutation in mitochondrial DNA associated with stroke‐like episodes and gastrointestinal dysfunction. Acta Neuropathol 2003; 105: 69–75 12471464 10.1007/s00401-002-0604-y

[61] Canafoglia L , Franceschetti S , Antozzi C , Carrara F , Farina L , Granata T , Lamantea E , Savoiardo M , Uziel G , Villani F , Zeviani M , Avanzini G . Epileptic phenotypes associated with mitochondrial disorders. Neurology 2001; 56: 1340–6 11376185 10.1212/wnl.56.10.1340

[62] Ikawa M , Yoneda M , Muramatsu T , Matsunaga A , Tsujikawa T , Yamamoto T , Kosaka N , Kinoshita K , Yamamura O , Hamano T , Nakamoto Y , Kimura H . Detection of preclinically latent hyperperfusion due to stroke‐like episodes by arterial spin‐labeling perfusion MRI in MELAS patients. Mitochondrion 2013; 13: 676–80 24095972 10.1016/j.mito.2013.09.007

[63] Iizuka T , Sakai F , Kan S , Suzuki N . Slowly progressive spread of the stroke‐like lesions in MELAS. Neurology 2003; 61: 1238–44 14610127 10.1212/01.wnl.0000091888.26232.fe

[64] Iizuka T , Sakai F , Suzuki N , Hata T , Tsukahara S , Fukuda M , Takiyama Y . Neuronal hyperexcitability in stroke‐like episodes of MELAS syndrome. Neurology 2002; 59: 816–24 12297560 10.1212/wnl.59.6.816

[65] Gropen TI , Prohovnik I , Tatemichi TK , Hirano M . Cerebral hyperemia in MELAS. Stroke 1994; 25: 1873–6 8073472 10.1161/01.str.25.9.1873

[66] Nishioka J , Akita Y , Yatsuga S , Katayama K , Matsuishi T , Ishibashi M , Koga Y . Inappropriate intracranial hemodynamics in the natural course of MELAS. Brain Dev 2008; 30: 100–5 17664050 10.1016/j.braindev.2007.06.008

[67] Peng NJ , Liu RS , Li JY , Tsay DG , Kong KW , Kwok CG , Strauss HW .Increased cerebral blood flow in MELAS shown by Tc‐99 m HMPAO brain SPECT. Neuroradiology 2000; 42: 26–9 10663465 10.1007/s002340050005

[68] Minobe S , Matsuda A , Mitsuhashi T , Ishikawa M , Nishimura Y , Shibata K , Ito E , Goto Y , Nakaoka T , Sakura H . Vasodilatation of multiple cerebral arteries in early stage of stroke‐like episode with MELAS. J Clin Neurosci 2015; 22: 407–8 25128282 10.1016/j.jocn.2014.05.021

[69] Rodan LH , Poublanc J , Fisher JA , Sobczyk O , Wong T , Hlasny E , Mikulis D , Tein I . Cerebral hyperperfusion and decreased cerebrovascular reactivity correlate with neurologic disease severity in MELAS. Mitochondrion 2015; 22: 66–74 25801712 10.1016/j.mito.2015.03.002

[70] Ito H , Mori K , Harada M , Minato M , Naito E , Takeuchi M , Kuroda Y , Kagami S . Serial brain imaging analysis of stroke‐like episodes in MELAS. Brain Dev 2008; 30: 483–8 18289816 10.1016/j.braindev.2008.01.003

[71] Ohshita T , Oka M , Imon Y , Watanabe C , Katayama S , Yamaguchi S , Kajima T , Mimori Y , Nakamura S . Serial diffusion‐weighted imaging in MELAS. Neuroradiology 2000; 42: 651–6 11071437 10.1007/s002340000335

[72] Tzoulis C , Bindoff LA . Serial diffusion imaging in a case of mitochondrial encephalomyopathy, lactic acidosis, and stroke‐like episodes. Stroke 2009; 40: e15–17 19095975 10.1161/STROKEAHA.108.523118

[73] Wang XY , Noguchi K , Takashima S , Hayashi N , Ogawa S , Seto H . Serial diffusion‐weighted imaging in a patient with MELAS and presumed cytotoxic oedema. Neuroradiology 2003; 45: 640–3 12898076 10.1007/s00234-003-1029-6

[74] Yoneda M , Maeda M , Kimura H , Fujii A , Katayama K , Kuriyama M . Vasogenic edema on MELAS: a serial study with diffusion‐weighted MR imaging. Neurology 1999; 53: 2182–4 10599803 10.1212/wnl.53.9.2182

[75] Graham BR , Pylypchuk GB . Posterior reversible encephalopathy syndrome in an adult patient undergoing peritoneal dialysis: a case report and literature review. BMC Nephrol 2014; 15: 10 24411012 10.1186/1471-2369-15-10 PMC3893488

[76] Beausang‐Linder M , Bill A . Cerebral circulation in acute arterial hypertension–protective effects of sympathetic nervous activity. Acta Physiol Scand 1981; 111: 193–9 7282395 10.1111/j.1748-1716.1981.tb06724.x

[77] Finsterer J , Barton P . Regression of stroke‐like lesions in MELAS‐syndrome after seizure control. Epileptic Disord 2010; 12: 330–4 21059492 10.1684/epd.2010.0338

[78] Bindoff LA , Engelsen BA . Mitochondrial diseases and epilepsy. Epilepsia 2012; 53(Suppl. 4): 92–7 22946726 10.1111/j.1528-1167.2012.03618.x

[79] Koga Y , Povalko N , Nishioka J , Katayama K , Kakimoto N , Matsuishi T . MELAS and L‐arginine therapy: pathophysiology of stroke‐like episodes. Ann N Y Acad Sci 2010; 1201: 104–10 20649546 10.1111/j.1749-6632.2010.05624.x

[80] Koga Y , Ishibashi M , Ueki I , Yatsuga S , Fukiyama R , Akita Y , Matsuishi T . Effects of L‐arginine on the acute phase of strokes in three patients with MELAS. Neurology 2002; 58: 827–8 11889254 10.1212/wnl.58.5.827

[81] Koga Y , Akita Y , Nishioka J , Yatsuga S , Povalko N , Tanabe Y , Fujimoto S , Matsuishi T . L‐arginine improves the symptoms of strokelike episodes in MELAS. Neurology 2005; 64: 710–12 15728297 10.1212/01.WNL.0000151976.60624.01

[82] Gilchrist JM , Sikirica M , Stopa E , Shanske S . Adult‐onset MELAS. Evidence for involvement of neurons as well as cerebral vasculature in strokelike episodes. Stroke 1996; 27: 1420–3 8711813 10.1161/01.str.27.8.1420

[83] Hasegawa H , Matsuoka T , Goto Y , Nonaka I . Strongly succinate dehydrogenase‐reactive blood vessels in muscles from patients with mitochondrial myopathy, encephalopathy, lactic acidosis, and stroke‐like episodes. Ann Neurol 1991; 29: 601–5 1892363 10.1002/ana.410290606

[84] Sakuta R , Nonaka I . Vascular involvement in mitochondrial myopathy. Ann Neurol 1989; 25: 594–601 2500889 10.1002/ana.410250611

[85] Betts J , Jaros E , Perry RH , Schaefer AM , Taylor RW , Abdel‐All Z , Lightowlers RN , Turnbull DM . Molecular neuropathology of MELAS: level of heteroplasmy in individual neurones and evidence of extensive vascular involvement. Neuropathol Appl Neurobiol 2006; 32: 359–73 16866982 10.1111/j.1365-2990.2006.00731.x

[86] Koga Y , Akita Y , Junko N , Yatsuga S , Povalko N , Fukiyama R , Ishii M , Matsuishi T . Endothelial dysfunction in MELAS improved by L‐arginine supplementation. Neurology 2006; 66: 1766–9 16769961 10.1212/01.wnl.0000220197.36849.1e

[87] Gierthmuhlen J , Allardt A , Sawade M , Wasner G , Baron R . Role of sympathetic nervous system in activity‐induced cerebral perfusion. J Neurol 2010; 257: 1798–805 20552364 10.1007/s00415-010-5613-5

[88] Iizuka T , Sakai F . Pathogenesis of stroke‐like episodes in MELAS: analysis of neurovascular cellular mechanisms. Curr Neurovasc Res 2005; 2: 29–45 16181098 10.2174/1567202052773544

[89] Turconi AC , Benti R , Castelli E , Pochintesta S , Felisari G , Comi G , Gagliardi C , Del Piccolo L , Bresolin N . Focal cognitive impairment in mitochondrial encephalomyopathies: a neuropsychological and neuroimaging study. J Neurol Sci 1999; 170: 57–63 10540037 10.1016/s0022-510x(99)00199-9

[90] Bosbach S , Kornblum C , Schroder R , Wagner M . Executive and visuospatial deficits in patients with chronic progressive external ophthalmoplegia and Kearns‐Sayre syndrome. Brain 2003; 126: 1231–40 12690061 10.1093/brain/awg101

[91] Neargarder SA , Murtagh MP , Wong B , Hill EK . The neuropsychologic deficits of MELAS: evidence of global impairment. Cogn Behav Neurol 2007; 20: 83–92 17558251 10.1097/WNN.0b013e3180335faf

[92] Kaufmann P , Shungu DC , Sano MC , Jhung S , Engelstad K , Mitsis E , Mao X , Shanske S , Hirano M , DiMauro S , De Vivo DC . Cerebral lactic acidosis correlates with neurological impairment in MELAS. Neurology 2004; 62: 1297–302 15111665 10.1212/01.wnl.0000120557.83907.a8

[93] Emmanuele V , Garcia‐Cazorla A , Huang HB , Coku J , Dorado B , Cortes EP , Engelstad K , De Vivo DC , Dimauro S , Bonilla E , Tanji K . Decreased hippocampal expression of calbindin D28K and cognitive impairment in MELAS. J Neurol Sci 2012; 317: 29–34 22483853 10.1016/j.jns.2012.03.005 PMC3345503

[94] Fan W , Waymire KG , Narula N , Li P , Rocher C , Coskun PE , Vannan MA , Narula J , Macgregor GR , Wallace DC . A mouse model of mitochondrial disease reveals germline selection against severe mtDNA mutations. Science 2008; 319: 958–62 18276892 10.1126/science.1147786 PMC3049809

[95] Iommarini L , Peralta S , Torraco A , Diaz F . Mitochondrial Diseases Part II: mouse models of OXPHOS deficiencies caused by defects in regulatory factors and other components required for mitochondrial function. Mitochondrion 2015; 22: 96–118 25640959 10.1016/j.mito.2015.01.008 PMC4447541

[96] Peralta S , Torraco A , Iommarini L , Diaz F . Mitochondrial Diseases Part III: therapeutic interventions in mouse models of OXPHOS deficiencies. Mitochondrion 2015; 23: 71–80 25638392 10.1016/j.mito.2015.01.007 PMC4516588

[97] Torraco A , Peralta S , Iommarini L , Diaz F . Mitochondrial Diseases Part I: mouse models of OXPHOS deficiencies caused by defects in respiratory complex subunits or assembly factors. Mitochondrion 2015; 21: 76–91 25660179 10.1016/j.mito.2015.01.009 PMC4364530

[98] Larsson NG , Wang J , Wilhelmsson H , Oldfors A , Rustin P , Lewandoski M , Barsh GS , Clayton DA . Mitochondrial transcription factor A is necessary for mtDNA maintenance and embryogenesis in mice. Nat Genet 1998; 18: 231–6 9500544 10.1038/ng0398-231

[99] Sorensen L , Ekstrand M , Silva JP , Lindqvist E , Xu B , Rustin P , Olson L , Larsson NG . Late‐onset corticohippocampal neurodepletion attributable to catastrophic failure of oxidative phosphorylation in MILON mice. J Neurosci 2001; 21: 8082–90 11588181 10.1523/JNEUROSCI.21-20-08082.2001 PMC6763882

[100] Dufour E , Terzioglu M , Sterky FH , Sorensen L , Galter D , Olson L , Wilbertz J , Larsson NG . Age‐associated mosaic respiratory chain deficiency causes trans‐neuronal degeneration. Hum Mol Genet 2008; 17: 1418–26 18245781 10.1093/hmg/ddn030 PMC2367695

[101] Ekstrand MI , Terzioglu M , Galter D , Zhu S , Hofstetter C , Lindqvist E , Thams S , Bergstrand A , Hansson FS , Trifunovic A , Hoffer B , Cullheim S , Mohammed AH , Olson L , Larsson NG . Progressive parkinsonism in mice with respiratory‐chain‐deficient dopamine neurons. Proc Natl Acad Sci USA 2007; 104: 1325–30 17227870 10.1073/pnas.0605208103 PMC1783140

[102] Good CH , Hoffman AF , Hoffer BJ , Chefer VI , Shippenberg TS , Backman CM , Larsson NG , Olson L , Gellhaar S , Galter D , Lupica CR . Impaired nigrostriatal function precedes behavioral deficits in a genetic mitochondrial model of Parkinson's disease. FASEB J 2011; 25: 1333–44 21233488 10.1096/fj.10-173625 PMC3058704

[103] de Haas R , Russel FG , Smeitink JA . Gait analysis in a mouse model resembling Leigh disease. Behav Brain Res 2015; 296: 191–8 26363424 10.1016/j.bbr.2015.09.006

[104] Quintana A , Kruse SE , Kapur RP , Sanz E , Palmiter RD . Complex I deficiency due to loss of NDUFS4 in the brain results in progressive encephalopathy resembling Leigh syndrome. Proc Natl Acad Sci USA 2010; 107: 10996–1001 20534480 10.1073/pnas.1006214107 PMC2890717

[105] Jain IH , Zazzeron L , Goli R , Alexa K , Schatzman‐Bone S , Dhillon H , Goldberger O , Peng J , Shalem O , Sanjana NE , Zhang F , Goessling W , Zapol WM , Mootha VK . Hypoxia as a therapy for mitochondrial disease. Science 2016; 352: 54–61 26917594 10.1126/science.aad9642 PMC4860742

[106] Peralta S , Torraco A , Wenz T , Garcia S , Diaz F , Moraes CT . Partial complex I deficiency due to the CNS conditional ablation of Ndufa5 results in a mild chronic encephalopathy but no increase in oxidative damage. Hum Mol Genet 2014; 23: 1399–412 24154540 10.1093/hmg/ddt526 PMC3929083

[107] Diaz F , Garcia S , Padgett KR , Moraes CT . A defect in the mitochondrial complex III, but not complex IV, triggers early ROS‐dependent damage in defined brain regions. Hum Mol Genet 2012; 21: 5066–77 22914734 10.1093/hmg/dds350 PMC3490513

[108] Weissbein U , Benvenisty N , Ben‐David U . Quality control: genome maintenance in pluripotent stem cells. J Cell Biol 2014; 204: 153–63 24446481 10.1083/jcb.201310135 PMC3897183

[109] Prigione A , Lichtner B , Kuhl H , Struys EA , Wamelink M , Lehrach H , Ralser M , Timmermann B , Adjaye J . Human induced pluripotent stem cells harbor homoplasmic and heteroplasmic mitochondrial DNA mutations while maintaining human embryonic stem cell‐like metabolic reprogramming. Stem Cells 2011; 29: 1338–48 21732474 10.1002/stem.683

[110] Hatakeyama H , Goto YI . Concise review: heteroplasmic mitochondrial DNA mutations and mitochondrial diseases: toward iPSC‐based disease modeling, drug discovery, and regenerative therapeutics. Stem Cells 2016; 34: 801–8 26850516 10.1002/stem.2292

[111] Hamalainen RH , Manninen T , Koivumaki H , Kislin M , Otonkoski T , Suomalainen A . Tissue‐ and cell‐type‐specific manifestations of heteroplasmic mtDNA 3243A>G mutation in human induced pluripotent stem cell‐derived disease model. Proc Natl Acad Sci USA 2013; 110: E3622–30 24003133 10.1073/pnas.1311660110 PMC3780874

[112] Folmes CD , Martinez‐Fernandez A , Perales‐Clemente E , Li X , McDonald A , Oglesbee D , Hrstka SC , Perez‐Terzic C , Terzic A , Nelson TJ . Disease‐causing mitochondrial heteroplasmy segregated within induced pluripotent stem cell clones derived from a patient with MELAS. Stem Cells 2013; 31: 1298–308 23553816 10.1002/stem.1389 PMC3706526

[113] Ma H , Folmes CD , Wu J , Morey R , Mora‐Castilla S , Ocampo A , Ma L , Poulton J , Wang X , Ahmed R , Kang E , Lee Y , Hayama T , Li Y , Van Dyken C , Gutierrez NM , Tippner‐Hedges R , Koski A , Mitalipov N , Amato P , Wolf DP , Huang T6 , Terzic A , Laurent LC , Izpisua Belmonte JC , Mitalipov S . Metabolic rescue in pluripotent cells from patients with mtDNA disease. Nature 2015; 524: 234–8 26176921 10.1038/nature14546

[114] Chung K , Deisseroth K . CLARITY for mapping the nervous system. Nat Methods 2013; 10: 508–13 23722210 10.1038/nmeth.2481

[115] Phillips J , Laude A , Lightowlers R , Morris CM , Turnbull DM , Lax NZ . Development of passive CLARITY and immunofluorescent labelling of multiple proteins in human cerebellum: understanding mechanisms of neurodegeneration in mitochondrial disease. Sci Rep 2016. 10.1038/srep26013 PMC4867607 27181107

